# Acetate: Friend or foe against breast tumour growth in the context of obesity?

**DOI:** 10.1111/jcmm.16034

**Published:** 2020-10-26

**Authors:** Caner Yelek, Lionel Mignion, Nicolas Joudiou, Romano Terrasi, Florian Gourgue, Matthias Van Hul, Nathalie Delzenne, Bernard Gallez, Cyril Corbet, Giulio G. Muccioli, Olivier Feron, Patrice D. Cani, Bénédicte F. Jordan

**Affiliations:** ^1^ Biomedical Magnetic Resonance Research Group Louvain Drug Research Institute UCLouvain Brussels Belgium; ^2^ Metabolism and Nutrition Research Group, WELBIO—Walloon Excellence in Life Sciences and BIOtechnology Louvain Drug Research Institute UCLouvain Brussels Belgium; ^3^ Nuclear & Electron Spin Technologies Platform Louvain Drug Research Institute UCLouvain Brussels Belgium; ^4^ Bioanalysis and Pharmacology of Bioactive Lipids Research Group Louvain Drug Research Institute UCLouvain Brussels Belgium; ^5^ Pole of Pharmacology and Therapeutics Institute of Experimental and Clinical Research UCLouvain Brussels Belgium

**Keywords:** acetate, high‐fat diet, hypoxia, metabolism, obesity, tumour growth

## Abstract

Acetate is reported as a regulator of fat mass but also as lipogenic source for cancer cells. Breast cancer is surrounded by adipose tissue and has been associated with obesity. However, whether acetate contributes to cancer cell metabolism as lipogenic substrate and/or by changing fat storage and eventually obesity‐induced breast cancer progression remains unknown. Therefore, we studied the contribution of acetate to breast cancer metabolism and progression. In vitro, we found that acetate is not a bioenergetic substrate under normoxia and did not result in a significant change of growth. However, by using lipidomic approaches, we discovered that acetate changes the lipid profiles of the cells under hypoxia. Moreover, while mice fed a high‐fat diet (HFD) developed bigger tumours than their lean counterparts, exogenous acetate supplementation leads to a complete abolishment of fat mass gain without reverting the HFD‐induced obesity‐driven tumour progression. In conclusion, although acetate protects against diet‐induced obesity, our data suggest that it is not affecting HFD‐driven tumour progression.

## 1 INTRODUCTION

Cancer is a disease characterized by rapid and uncontrolled division of mutated cells in the body. This abnormal proliferation is accompanied by higher metabolic requirements in order to supply enough anabolic precursors (i.e. lipids, amino acids and nucleotides).^1^ Aside from the oncogenic mutations favouring one or the other metabolic pathway, the tumour microenvironment (TME) of solid tumours also plays an important role in dictating the metabolic activity of cancer cells. One of those factors is hypoxia, mainly caused by deficient vascularity and high consumption of oxygen.^2^ It has been reported that hypoxia promotes a lipogenic cancer cell phenotype via both HIF‐1ɑ dependent and independent mechanisms.^3^ Furthermore, Schug and colleagues highlighted a higher sensitivity to fatty acid synthesis inhibition under low serum and oxygen availability, underlining the crucial role of lipid synthesis for cancer cell survival and growth under these circumstances.^4^

The sole precursor for lipid synthesis is cytosolic acetyl‐CoA, as mitochondrial production is directed towards the tricarboxylic acid (TCA) cycle. The lipogenic acetyl‐CoA emerges from two distinct sources, either by ATP citrate lyase (ACLY) or acetyl‐CoA synthetase 2 (ACSS2) activity. The first enzyme catalyses the conversion of citrate to acetyl‐CoA, and the latter produces acetyl‐CoA from the short‐chain fatty acid (SCFA) acetate.^5^ Kamphorst and colleagues demonstrated by using ^13^C‐acetate tracing experiments that acetate significantly contributed to the pool of lipogenic acetyl‐CoA in an in vitro model of human breast cancer under hypoxia.^6^ Two other studies reported, under a metabolic stress like hypoxia and/or low serum, an increased demand for lipogenic acetyl‐CoA supplied mainly through glutamine and acetate metabolism. Additionally, they noted an increase of ACSS2 expression and a decrease in cell proliferation following ACSS2 depletion under hypoxia. This observation correlates with a higher uptake of acetate in breast cancer models, highlighting the necessity of ACSS2 for acetate uptake.^4^, ^7^

Apart from its lipogenic function, ACSS2 is necessary for the recapture of endogenously produced acetate by histone deacetylase activity.^7^, ^8^ In addition, knock‐down of ACSS2 diminished tumour burden and growth in vivo in various models including colon, liver, skin and breast cancer. Likewise, Gao and colleagues demonstrated that combining hypoxia and acetate increased the acetylation of histones in the promoter regions of lipogenic genes (i.e. *Fasn*, *Acaca*, *Acss2*) resulting in their higher expression.^9^ In contrast to its role in lipogenesis, acetate has been reported as a significant contributor to the TCA cycle and oxidative phosphorylation in glioblastoma and brain metastases.^10^ This contradiction underlines that the fate of acetate is dependent on the cancer type and that it can be used for both lipogenesis and ATP production.

Besides its contribution to cancer metabolism, acetate has profound effects on whole‐body metabolism and fat mass.^11^ Along these lines, obesity has been associated with the development and progression of certain types of cancers including, but not limited to, gastrointestinal cancers, prostate, ovarian and particularly breast cancer.^12^, ^13^, ^14^ Most of the associations point to the peritumoural adipose tissue, another key component of the TME, as a source of nutrient and support for cancer cell growth and progression.^15^, ^16^, ^17^, ^18^ Interestingly, two independent studies revealed that mice fed a high‐fat diet (HFD) supplemented with acetate exhibited a limited bodyweight gain compared to mice fed only with HFD.^19^, ^20^ However, whether acetate contributes to cancer cell metabolism as lipogenic substrate and/or by changing fat mass storage and eventually obesity‐induced breast cancer progression remains unknown.

Therefore, we explored this question by using both in vitro and in vivo approaches. First, we investigated the direct contribution of acetate to breast cancer cell metabolism as a bioenergetic and a lipogenic substrate under normoxic and hypoxic conditions in vitro. Then we analysed whether acetate could give a proliferative advantage in vitro and in vivo. Finally, we examined whether obesity indeed induced a faster breast tumour growth and if the protective effects of acetate against obesity could be beneficial in the context of obesity‐driven tumour growth in the C57BL/6 mouse strain which is sensitive to diet‐induced obesity.^21^

## 2 METHODS

### 2.1 Cell culture and reagents

PY230 *Mus musculus* mammary gland adenocarcinoma cell line was acquired from American Type Cell Culture (ATCC) and stored according to the supplier's instructions. Cells were maintained in culture in F‐12K medium (GIBCO) supplemented with 5% heat‐inactivated FBS (Thermo Fisher Scientific) and 0.1% Mito+Serum Extender (Fisher Scientific) in a humidified atmosphere at 37°C and 5% CO_2_. Sodium acetate (Sigma Aldrich) was directly dissolved in the culture medium. All in vitro experiments were performed in F‐12K medium (GIBCO) supplemented with 10% heat‐inactivated dialysed FBS (Thermo Fisher Scientific) in a humidified atmosphere at 37°C, 5% CO_2_ and 20% or 1% O_2_.

### 2.2 Cell density

Cell density was assessed using the PrestoBlue reagent (Thermo Fisher Scientific) according to the manufacturer's instruction. Briefly, cells were treated with indicated concentrations of sodium acetate for 24 hours before addition of 10% PrestoBlue Reagent. After 2 hours of incubation, fluorescence intensity was measured (λex/λem = 560/590 nm) using a plate reader (SpectraMax M2e, Molecular Devices). All data were normalized to the fluorescence intensity of untreated wells and expressed in per cent of control.

### 2.3 Cell proliferation

Cell proliferation was measured by using a 5‐bromo‐2‐deoxyuridine (BrDu) incorporation ELISA‐based kit (Roche) following the provider's protocol. After 24 hours of incubation with indicated concentrations of sodium acetate, BrDu was added to the culture medium for 2 hours. Then, after a fixation step of the cells with a solution provided by the manufacturer and binding of anti‐BrDu antibody coupled to a peroxidase, cellular proliferation was assessed by measuring the absorbance at 370 nm using a plate reader (SpectraMax M2e, Molecular Devices). All data were normalized to the absorbance of untreated wells and expressed in per cent of control.

### 2.4 Oxygen consumption rate with Seahorse XF96 analyser

Oxygen consumption rate (OCR) was determined using a Seahorse XF96 analyser (Agilent Technologies). Cells were seeded in culture medium as described above. The day after, the medium was changed to a basal DMEM medium with no buffer, serum, glucose nor glutamine and adjusted to pH 7.4. After one hour of incubation at 37°C without CO_2_, basal respiration rate was measured three times before addition of acetate dissolved in the same medium for a final indicated concentration. The oxygen consumption rate was then measured five times to assess the oxygen consumption rate induced by acetate.

### 2.5 Oxygen consumption rate by electron paramagnetic resonance

The effect of acetate on OCR of PY230 cell line was evaluated via an X‐band electron paramagnetic resonance (EPR) spectrometer operating at 9 GHz (Bruker EMX plus) as previously described.^22^ Cells were incubated with determined concentrations of acetate for 24 hours, and after trypsinization, cells were resuspended in F‐12K medium containing acetate with 10% FBSD at a concentration of 2 million cells/mL. The cell suspension was then mixed 1:1 with 20%w/v of dextran in PBS solution and ^15^N‐PDT at a final concentration of 5.5 µM as an oxygen sensor. The cell suspension was then introduced to a glass capillary tube (Hirschmann Labogeräte) sealed and inserted into a quartz tube then placed into the EPR cavity. The cavity was continuously flushed with N_2_ gas mixture (400 L/h) at 37°C throughout the acquisition. EPR spectra were acquired every minute, and pO2 values were obtained by measuring the peak‐to‐peak EPR signal linewidths. The linewidths were then converted into pO2 by means of a calibration curve. OCR was then calculated as the slope of pO2 over time curve.

### 2.6 Western blot

Whole cell lysates were obtained with RIPA lysis buffer (Thermo Fisher Scientific) complemented with 1% halt protease inhibitor and 3% halt phosphatase inhibitor (Thermo Fisher Scientific) and mixed with Laemmli buffer. The mix was heated in a dry bath at 95°C for 7 minutes for further denaturation. Equal amounts of proteins were loaded and separated by SDS‐PAGE in TGS buffer (Bio‐Rad). Proteins were then transferred on a PVDF membrane (Bio‐Rad RTA transfer kit) on a semi‐dry transfer system (Bio‐Rad). Non‐specific binding sites on the membrane were blocked with TBS buffer with 0.1% tween and 5% milk. After an overnight incubation with primary antibodies in TTBS‐milk 1% (ACSS2 1:1000; Abcam ab66038, ß‐actin 1:1000; Cell Signaling Technology 4970S) and incubation with secondary antibody the day after, the protein bands were detected with SuperSignal West Pico Plus (Thermo Fisher Scientific).

### 2.7 Lipidomic analysis

Lipid content of PY230 cells line was characterized by liquid chromatography coupled to a mass spectrometer. Lipid species were analysed after liquid/liquid extraction and solid phase extraction purification in the presence of internal standards. Lysophospholipids, phospholipids and sphingomyelins were analysed using a LTQ‐Orbitrap mass spectrometer coupled to an Accela LC system (Thermo Fischer Scientific). For this analysis, we used a Kinetex LC‐18 (150 × 4.6 mm, 5 μm) column (Phenomenex) and a gradient between phase A (Methanol‐Acetonitrile (9:1, v/v) 75%, H_2_O 25% containing 5 mM ammonium acetate), phase B (Methanol‐Acetonitrile (9:1, v/v) containing 5 mM ammonium acetate) and phase C (isopropanol containing 5 mM ammonium acetate). The gradient (400 μL/min) increased linearly from 100% A to 100% B in 15 minutes and after 10 minutes from 100% B to 70% B ‐ 30% C over 5 minutes. This was maintained over 30 minutes before equilibrating the system. The lipids were analysed in negative mode using an electrospray ionization probe.^23^ Ceramides were analysed using a LTQ‐Orbitrap mass spectrometer coupled to an Accela LC system (Thermo Fischer Scientific) as previously reported.^24^ We used a Poroshell 120 LC‐18 (150 × 4.6mm, 4 μm) column (Agilent) and a gradient between phase A (Methanol‐H_2_O (75:25, v/v) containing 0.1% of acetic acid) and phase B (Methanol containing 0.1% acetic acid). The gradient (400 μL/min) increased linearly from 100% A to 100% B over 15 minutes and was hold at 100% for an additional 75 minutes before equilibrating the system. The flow was switched from 400‐600 µL/min after 30 minutes. These lipids were analysed in positive mode using an APCI probe. For all lipid species, the relative quantification was based on the ratio of area under the curve (AUC) of the lipid of interest and the AUC of the respective internal standard.

### 2.8 Mice

All mouse experiments were approved by the ethical committee for animal care of the Health Sector of the Université catholique de Louvain, under the supervision of JP Dehoux, under the specific number 2017/UCL/MD/005 and performed in accordance with the guidelines of the local ethics committee and in accordance with the Belgian Law of 29 May 2013, regarding the protection of laboratory animals (agreement number LA1230314). All mice (Janvier Labs) were 8‐week‐old female‐specific pathogen‐free C57BL/6JRj mice at the beginning of the experiments. Cages were randomly assigned to experimental groups to ensure that each group was matched in terms of bodyweight at the beginning of normal diet (ND) and high‐fat diet (HFD) feeding but also before the assignment of the associated treatment. All mice were housed in specific pathogen‐free conditions and in a controlled environment (room temperature of 23 ± 2°C, 12 hours daylight cycle) with ad libitum access to irradiated food (either AIN93Mi for ND or D12492i for HFD; Research diet) and sterile water. Bodyweight and body composition (lean and fat mass) were assessed weekly, when necessary for the experiment, by using 7.5 MHz time domain‐nuclear magnetic resonance (TD‐NMR; LF50 minispec, Bruker).

### 2.9 Tumour growth experiments and tissue sampling

Tumours were induced by subcutaneous injection of 2 × 10^6^ PY230 cells in the 5th mammary fat pad of C57Bl/6 female mice. The cells were freshly passaged just before the injection and prepared as a mixture of 1:1 PBS & Matrigel (Corning) and injected within 30 minutes. The treatment of the mice began the day after the tumour inoculation. 300 mM sodium acetate (Sigma) and pH‐matched control solutions were freshly prepared in sterile water and renewed every week. Tumour sizes were monitored at least twice a week and measured using an electronic caliper by an independent experimenter. At the end of the experiment, mice were anesthetized with isoflurane after a fasting period of 6 hours. Blood was sampled from the portal and cava veins. After blood sampling, mice were killed by cervical dislocation. Tissues were precisely dissected, weighed and immediately immersed in liquid nitrogen followed by storage at −80°C for further analysis.

### 2.10 RNA extraction and RT‐qPCR analysis

Total RNA was prepared from tissues using TriPure reagent (Roche). Quantification and integrity analysis of total RNA were performed by analysing 1 μL of each sample in an Agilent 2100 Bioanalyzer (Agilent RNA 6000 Nano Kit). cDNA was prepared by reverse transcription of 1 μg total RNA using a Reverse Transcription System kit (Promega). Real‐time quantitative PCR was performed with QuantStudio™ 3 Real‐Time PCR System (Thermo Fisher Scientific). All samples were performed in duplicate, and data were analysed according to the 2^−ΔΔCT^ method. The identity and purity of the amplified product were assessed by melting curve analysis at the end of amplification. The chosen housekeeping gene is *Rpl19*.

*Rpl19* forward: GAAGGTCAAAGGGAATGTGTTCA; reverse: CCTTGTCTGCCTTCAGCTTGT, *Fasn* forward: CAGGCCCCTCTGTTAATTGG; reverse: TCCAGGGATAACAGCACCTT, *Cpt1a* forward: AGACCGTGAGGAACTCAAACCTAT; reverse: TGAAGAGTCGCTCCCACT.

### 2.11 Triglycerides and non‐esterified fatty acids quantification

Triglycerides and NEFAs were assayed with their respective kits (Randox) according to the manufacturer's instructions. Briefly, blood samples coming from the vena cava were mixed with the reagents from the kits and quantified by measure of absorbance with a plate reader at 500 and 550 nm (Spectramax i3).

## 3 RESULTS

### 3.1 Exogenous acetate does not benefit PY230 cells under normoxia in vitro but lowers cell density and induces changes in cell lipid profiles under hypoxia

**Figure 1:**
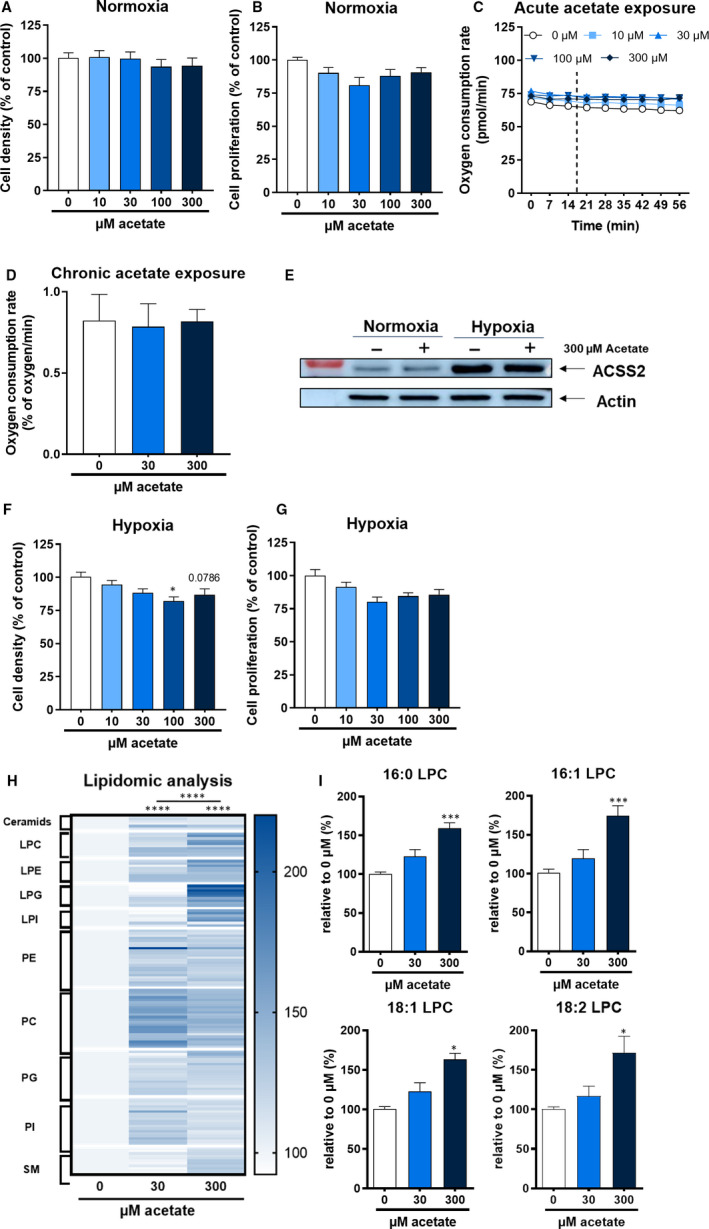
Exogenous acetate does not benefit PY230 cells under normoxia in vitro but lowers cell density and induces changes on PY230 cells lipid profiles under hypoxia. (A) Cell density and (B) proliferation of PY230 cells after 24 hours of treatment with increasing concentrations of sodium acetate under normoxia. (C) Cellular respiration measured by Seahorse respirometry after acute addition of acetate at indicated concentrations in the culture medium. (D) Cellular respiration measured by EPR after 24 hours of exposure to acetate before and during the acquisition at indicated concentrations. (E) Western blot against ACSS2 after 48 hours of exposition to hypoxia in presence or absence of acetate. (F) Cell density and (G) proliferation of PY230 cells after 24 hours of treatment with increasing concentrations of sodium acetate under hypoxia. (H) Lipidomic heatmap representing the different lipid families and their mean relative quantification upon 48 hours of acetate treatment under hypoxia. Abbreviations: L: (Lyso‐); P (Phosphatidyl‐); C: (Choline); E: (Ethanolamine); G: (Glycerol); I: (Inositol); SM: (Sphingomyelin). (F) LPC and LPE quantification expressed relatively to the control. All data are expressed as mean ± SEM of three independent experiments. Statistical analysis: (A, B, D, F, G, I) Nested one‐way ANOVA followed by Dunnett's multiple comparison test. (H) One‐way ANOVA followed by Tukey's multiple comparison test: *P < .05; **P < .01; ***P < .001; ****P < .0001

The short‐chain fatty acid acetate has been described as a bioenergetic substrate^10^ and a lipogenic substrate under metabolic stress conditions such as hypoxia.^4^, ^6^, ^7^, ^8^ Thus, we first studied in vitro whether the PY230 triple‐negative breast cancer cell line could benefit from an exogenous supplementation of acetate for its growth. Within a physiological range of acetate concentration,^25^, ^26^, ^27^ we observed a lack of significant change of cell density under normoxia (Figure 1A). Similar results have been obtained regarding the cell proliferation, as assessed by the BrDu incorporation assay (Figure 1B), suggesting that acetate does not give a proliferative advantage under normoxic conditions. We then evaluated the contribution of acetate as a fuel for cellular respiration and found that exposing the cells to acetate did not induce further increase in cellular respiration (Figure 1C). Additionally, we examined the oxygen consumption rate of cells cultured in the presence of acetate for 24 hours. Yet again, the presence of exogenous acetate in the culture medium did not increase the oxygen consumption rate (Figure 1D), pointing that acetate the supplemented acetate is not fuelling the TCA cycle. Taken altogether, these data show that exogenous acetate does not benefit PY230 cells under normoxia in vitro.

Because it has also been described that acetate could profit cancer cells by being a lipogenic substrate under hypoxia mainly through an ACSS2‐dependent mechanism, we verified in our model if ACSS2 expression increased under hypoxia. Indeed, there was a significant increase of the expression of ACSS2 under hypoxia regardless of the presence of acetate (Figure 1E). Next, we evaluated in vitro the effect of acetate on cell density under hypoxia within the concentration ranges described previously. Surprisingly, acetate slightly decreased the cell density in a dose‐dependent manner (Figure 1F). Although not statistically significant, the same trend was observed for cellular proliferation (Figure 1G), suggesting that, in contradiction with what was described previously, acetate might inhibit cellular proliferation. Furthermore, in order to investigate changes on the cell lipid profiles upon addition of exogenous acetate under hypoxia, we conducted a lipidomic analysis by LC/MS‐MS. We found an increase in the total amount of lipids after an acetate treatment, indicating a higher lipid content (Figure 1H). Among the lipid families, lysophosphatidylcholines (LPC) were the most significantly increased (Figure 1I), more precisely saturated and unsaturated LPCs with a chain‐length of 16 carbons.

### 3.2 Exogenous acetate supplementation does not influence tumour growth in vivo

**Figure 2:**
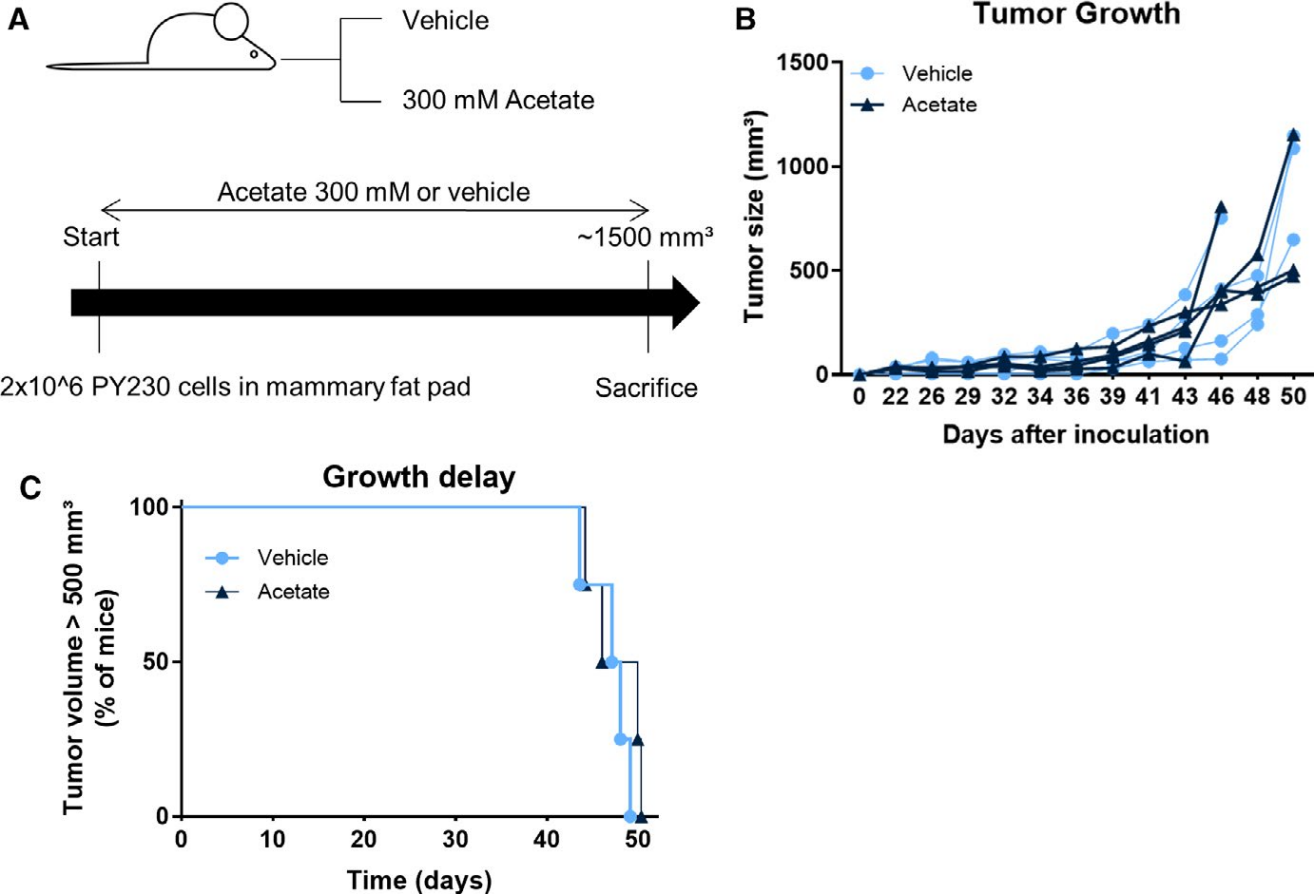
Exogenous acetate supplementation does not influence tumour growth in vivo. (A) Experimental design (N = 4 mice for each group). (B) Tumour size evolution over time for each mouse. The mice have been given 300 mM acetate or pH‐matched drinking water. (C) Kaplan‐Meier analysis of tumour growth with 500 mm^3^ as end‐point

Given that acetate lowers cell density under hypoxia and induces specific changes on the lipid profile under this condition, we conducted an in vivo experiment to study if an exogenous intake of acetate could influence in vivo tumour growth (Figure 2A). We investigated tumour growth in mice treated with either 300 mM of sodium acetate or vehicle. Mice were sacrificed when the tumour size reached a critical end‐point. We observed that exogenous acetate had no influence, neither on tumour size, nor on tumour growth rate (time to reach 500 mm^3^) (Figure 2B,C), indicating that acetate is not utilized by our tumour model in vivo. Taken collectively with the in vitro data, we can conclude that exogenous acetate supplementation does not influence or feed tumour growth and metabolism in vitro and in vivo in our model of murine triple‐negative breast cancer.

### 3.3 Acetate‐induced fat mass loss does not counteract obesity‐driven tumour progression

**Figure 3:**
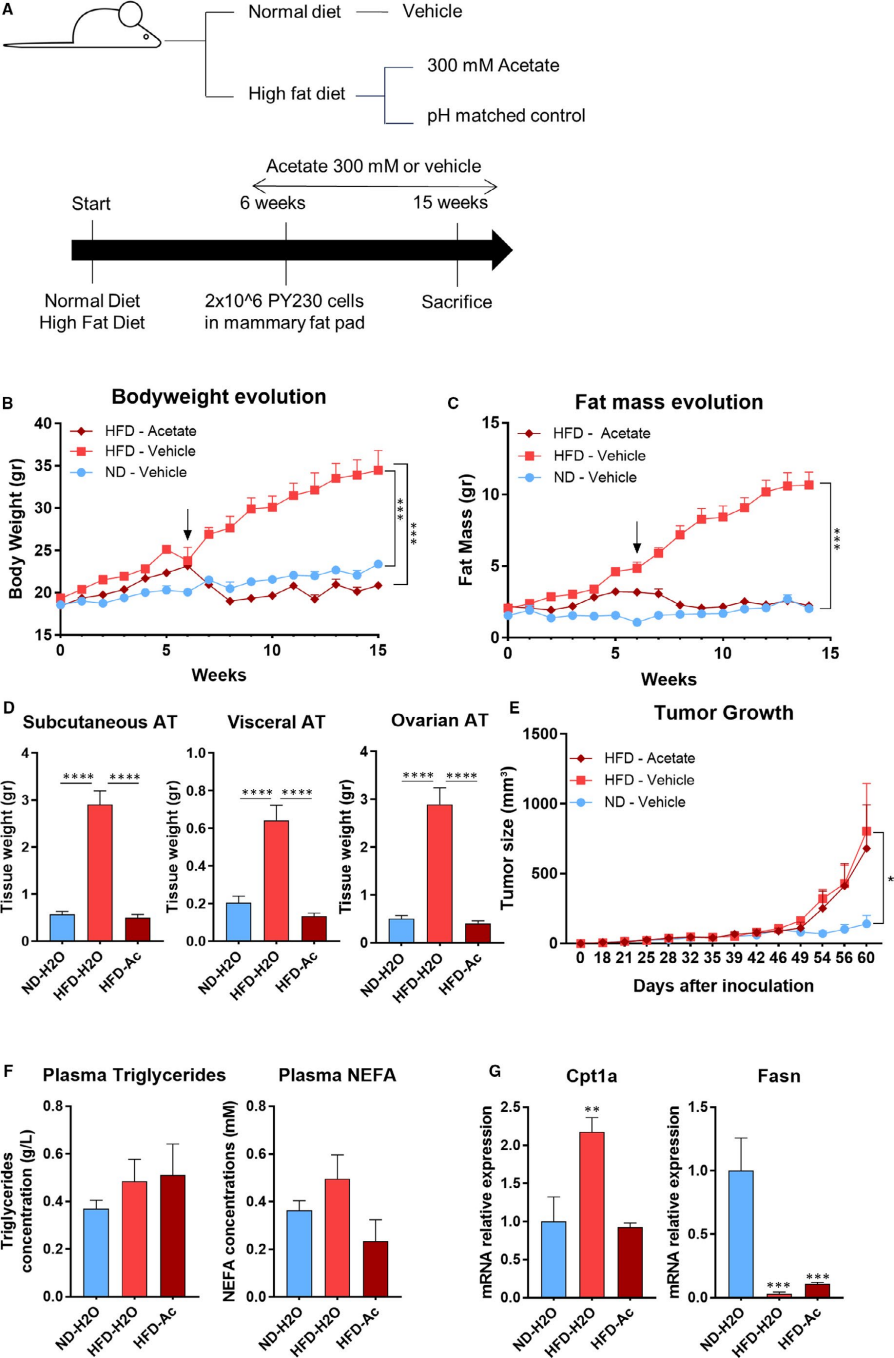
Acetate‐induced fat mass loss does not counteract obesity‐driven tumour progression. (A) Experimental design. (ND‐H_2_O: N = 4; HFD‐H_2_O and HFD‐Ac: N = 5 mice for both groups). (B) Bodyweight and (C) fat mass evolution of mice during the experiment ND = normal diet, HFD = high‐fat diet. Mice have been treated either with 300 mM acetate or pH‐matched drinking water. (D) Weight of representative adipose tissues (AT) at the necropsy. (E) Tumour size evolution over time. (F) Plasma triglycerides (TG) and non‐esterified fatty acids (NEFA) of fasting mice sampled at the necropsy. (G) mRNA expression in the subcutaneous adipose tissue quantified by RT‐qPCR. All data are expressed as mean ± SEM. Statistical analysis: (B‐C) two‐way ANOVA followed by Tukey's multiple comparison test. (D‐F‐G) One‐way ANOVA followed by Tukey's multiple comparisons test. (E) Two‐way ANOVA followed by Dunnett's multiple comparison test calculated after log transformation of the data. *P < .05; **P < .005; ***P < .0005

Acetate has been described as a strong regulator of bodyweight in rodent models fed a diet supplemented with diverse SCFAs including acetate.^19^, ^20^ In addition, it has been reported that obesity promotes tumour progression.^17^, ^28^ Thus, we investigated whether acetate supplementation could be beneficial against tumour growth in the context of obesity by supplementing acetate to high‐fat diet fed mice in which tumour cells were injected (Figure 3A). As expected, HFD‐fed mice gained significantly more bodyweight and fat mass than their normal diet (ND) counterparts (Figure 3B‐D). This difference was completely abolished in HFD‐fed for which the drinking water was mice supplemented with acetate (Figure 3B). This was associated with a complete abolishment of HFD‐induced adipose tissue development, with fat depots reduced to similar levels as the ND‐fed mice (Figure 3C,D).

As expected, obese mice developed bigger tumours than their leaner counterparts. Strikingly, although displaying a strongly reduced bodyweight and fat mass, the acetate‐treated HFD‐fed mice had tumours within the same size range as those of the obese mice (Figure 3E). We measured plasma triglycerides (TG) and non‐esterified fatty acid (NEFA) levels of these mice. Although not statistically significant, plasma TG levels of both HFD groups were higher than the ND group (Figure 3F). However, plasma NEFA levels were lower in acetate‐treated HFD group compared to both ND and HFD group (Figure 3F). These results underline that there is enough supply of fatty acids, coming mainly from the diet and less from the adipose tissue, to the tumour under HFD even when the mice are lean. Finally, we measured mRNA expression of genes involved in fatty acid oxidation (*Cpt1a*) and lipogenesis (*Fasn*) in the subcutaneous adipose tissue. Our results show that under HFD there is a strong decrease of *Fasn* expression regardless of the group and lower *Cpt1a* expression upon acetate treatment (Figure 3G). These results indicate that acetate‐induced fat mass loss is not sufficient to counteract obesity‐driven tumour progression.

## 4 DISCUSSION

In this study, we have shown that exogenous acetate is not used as a bioenergetic substrate for breast cancer cell. Although we found that cells exposed to acetate exhibited a distinct lipid profiles when exposed to hypoxia, exogenous acetate supplementation did not significantly affect cells growth in vitro and in vivo. In addition, while HFD‐fed mice developed larger tumours than their lean counterparts, acetate supplementation did not affect tumour progression despite completely blocking HFD‐induced fat mass gain.

To date, only one study conducted by Mashimo and colleagues demonstrated the contribution of acetate to the TCA cycle in glioblastoma and brain metastases, suggesting that this metabolic pathway might be specific to tumour growth occurring in the central nervous system.^10^ Indeed, the metabolic phenotype of tumours is highly dependent of both their tissue of origin and their nurture.^1^, ^29^ Hence, acetate oxidation might not be needed as an energetic source in this model.

However, lipidomics data revealed modest but significant increases of lipids and changes in cell lipid profiles upon an acetate treatment under hypoxia, specifically lipids from the LPC family thereby suggesting an increased lipid synthesis as described previously.^6^, ^9^ Lysophosphatidylcholines are bioactive lipids associated with an increase of inflammatory markers in various tissues.^30^ LPCs have been shown to be increased under hypoxia.^31^ In our study, this increase seemed to be further amplified upon acetate treatment. They have also been described as a more accessible source of nutrient for cells under hypoxia.^32^ Nevertheless, these changes are not correlated to a change in the proliferative phenotype of the cells in vitro. Furthermore, LPCs have been characterized to act as pro‐metastatic factors by increasing cell motility and adhesion in a model of rhabdomyosarcoma.^33^ Even though this aspect remains unknown, the increase (around 60%) might be too small to induce any noticeable change in cell motility and migration.

Aside from its impact on cellular metabolism, we explored the potential benefit of exogenous acetate on tumour growth in vitro and in vivo. Our in vitro results showed that addition of acetate to the culture medium does not induce significant change in cellular proliferation under normoxia but a slight decrease under hypoxia. These results might seem contradictory to what was reported previously ^4^, ^8^, ^34^ but most of the studies focused on ACSS2 rather than exogenous acetate per se. In fact, they demonstrated the crucial role of ACSS2 for cellular and tumour growth under stress conditions by using knock‐down or knockout experiments.^8^, ^34^ Besides its role for the uptake and transformation of acetate to acetyl‐CoA, ACSS2 also participates in histone acetylation and ensures that histone acetylation levels remain adequate under hypoxia.^7^, ^8^, ^9^ Thus, ACSS2 could act more as a co‐factor or as a transcription factor rather than a metabolic enzyme.^35^, ^36^

Although we observed an increase in ACSS2 expression under hypoxia, the addition of acetate did not further enhance the cellular proliferation. It is worth noting that acetate could also be produced endogenously from glucose by the activity of pyruvate dehydrogenase and H_2_O_2._ Therefore, exogenous acetate uptake might not be mandatory for the cell metabolism.^37^ We may speculate that the modest decrease in cell density could be explained by the activation of the acetate receptor GPR43. Indeed, acetate has been shown to reduce cellular proliferation and induce cell death in cancer cells from colorectal cancer and the human breast cancer cell line MCF‐7 by a GPR43‐dependent mechanism, although at concentrations much higher than those used in the current study,.^38^, ^39^, ^40^ However, we did not observe any effects of acetate supplementation on tumour growth in vivo. This lack of effect is not likely to be due to the dose administered as we found that acetate completely abolished HFD‐induced bodyweight and fat mass gain, an observation which is in accordance with previous studies.^19^, ^20^

Consistent with previous reports,^17^, ^28^ obese mice developed bigger tumours than their lean counterparts. Although acetate supplementation normalized bodyweight and the fat mass, it did not revert HFD‐driven tumour progression. Yet, even if these mice display a normal bodyweight, they had similar circulating levels of triglycerides to obese mice. This discrepancy could explain why acetate‐supplemented mice had tumours of the same size as those of HFD‐fed mice. Indeed, high circulating triglyceride levels might supply enough lipids to the tumour and fuel its growth. An element supporting this hypothesis is the considerable drop in *Fasn* expression upon an HFD in subcutaneous adipose tissue, an effect which is not rescued by acetate treatment. In our case, the main source of lipids is likely the diet rather than the adipose tissue. Altogether, these observations highlight the importance of lipid metabolism for tumour growth ^41^, ^42^ regardless of its source.

In conclusion, the short‐chain fatty acid acetate shows no benefit neither as a fuel nor as a growth‐promoting factor. Moreover, acetate‐promoted fat mass loss does not improve the outcome of tumour growth under a high‐fat diet.

## CONFLICT OF INTEREST

The authors declare that they have no conflicts of interest.

## AUTHOR CONTRIBUTIONS

**Caner Yelek:** Conceptualization (equal); Formal analysis (equal); Investigation (equal); Methodology (equal); Writing – original draft (equal). **Lionel Mignion:** Investigation (equal); Methodology (equal); Resources (equal). **Nicolas Joudiou:** Investigation (equal); Methodology (equal); Resources (equal). **Romano Terrasi:** Investigation (equal); Methodology (equal). **Florian Gourgue:** Methodology (equal); Resources (equal). **Matthias Van Hul:** Methodology (equal); Resources (equal); Writing – review and editing (equal). **Nathalie Delzenne:** Resources (equal). **Bernard Gallez:** Resources (equal). **Cyril Corbet:** Resources (equal); Supervision (equal); Writing – review and editing (equal). **Giulio M Muccioli:** Methodology (equal); Resources (equal); Writing – review and editing (equal). **Olivier Feron:** Conceptualization (equal); Resources (equal); Supervision (equal); Writing – review and editing (equal). **Patrice D Cani:** Conceptualization (equal); Funding acquisition (equal); Supervision (equal); Writing – review and editing (equal). **Benedicte F Jordan:** Conceptualization (equal); Funding acquisition (equal); Supervision (equal); Writing – review and editing (equal).

## Data Availability

The data that support the findings of this study are available from the corresponding author upon reasonable request.
